# Effect of exergaming on wellbeing of residents in a nursing home: a single blinded intervention study

**DOI:** 10.1007/s40520-021-01903-1

**Published:** 2021-06-22

**Authors:** Marlies Gunst, Isabelle De Meyere, Hannah Willems, Birgitte Schoenmakers

**Affiliations:** Department of Public Health and Primary Care, KU Leuven, Kapucijnenvoer 33, 7001, 3000 Leuven, Belgium

**Keywords:** Exergames, Older persons, Nursing home, Care quality

## Abstract

**Introduction:**

To improve the quality of life in nursing homes, meaningful activities and social contact are indispensable. Exergames can play a role addressing these needs.

**Methods:**

In a randomized single blinded controlled intervention study, we investigated the effect of playing exergames on general wellbeing, fun and on social interaction.

**Results:**

Thirty-five residents participated: 18 residents took part in the intervention group and 17 in the control group. The median mental wellbeing score of the intervention group increased from 42/50 to 45. The median sleep score of the intervention increased from 23/30 to 28. The median pain score of the intervention group improved from 18/20 to 20. The median score on subjective cognition increased from 24/30 to 26 while the mean scores on the objective assessment decreased from 1.8/2 to 1.7. Coaches gave an average fun score of 8.9/10 and an average intensity of exercise score of 11.6/20. Residents and coaches appreciated the social contact. Coaches reported a high feasibility (average of 4.1/5) but a low accessibility and a high intensity of supervision.

**Conclusions:**

Exergaming is a feasible and pleasant complement to the usual activities with a positive impact on wellbeing, sleep, pain, and perceived cognition. Future research should focus on vulnerable groups and aim to develop a study in an implementation design.

## Introduction

The society is dealing with an enormous aging of the population. According to a report by the Federal Service of Care and Health, 1.2% of the Flemish population lived in residential care in the period from 2009 to 2016 [1]. In Belgium, the current share of the age group 65–79 will increase from 13 per 1000 inhabitants to 179 per 1000 inhabitants by 2030. In December 2017, the Flemish Minister of Welfare, Public Health and Family published a report in which more than 20,000 residents in Flemish residential care centres gave their opinion on various aspects that contribute to quality of life [2]. The main point of action turned out to be providing a meaningful daily activity and maintaining interpersonal emotional relationships.

Beside the traditional activities, exergames could fulfil both working points [3, 4]. Exergames are video games played by moving the body, combining physical and cognitive activity with social interaction. Movements are registered via a camera and an avatar will imitate the movements on a screen. It appears to the person who is moving, as if he is practicing that particular sport.

Exercise programs have already shown in the past that having a beneficial effect on falls, allowing the older person to be autonomous and independent for a longer period [5, 6]. These authors found that muscle strength, balance, walking, and reaction time significantly improved after several weeks of exergaming. An internal report from a residential care centre shows that self-reliance improved in half of the participants playing exergames [7].

A meta-analysis compared 17 RCTs examining the effect of exergames on memory and cognition and concluded that exergames have a positive effect on global cognition [8]. Other research showed that when physical activity was combined with cognitive exercise, additional effects occurred on different cognitive domains [8–10]. In 2010, Kemperman et al. suggested that the combination of physical and cognitive activity positively impacted memory through a process called “brain plasticity” [11]. Due to the virtual character of exergames and the direct feedback on movements performed by the gaming person, a positive effect is observed in the visual memory and reaction time [8].

Above, the psychosocial wellbeing of older people with dementia improved after playing exergames [3, 12]. In particular, quality of life, feelings of autonomy and competence increased. Sleep is considered an important prerequisite for maintaining health and general wellbeing. The process of “brain plasticity” described above takes place during deep sleep. In a review, the beneficial effect of sleep on long-term memory was confirmed [13]. Physical activity and cognitive exercise both have positive effects on sleep [14, 15].

In a randomized controlled single blinded intervention study, we investigated the effect of playing exergames on four domains of wellbeing in older people living in a residential care centre: mental wellbeing, sleep quality, pain complaints, and cognitive functioning. In the secondary research question, we investigated if the elderly enjoyed playing the exergames and if social interaction improved through gaming. Finally, we investigated the feasibility of integrating these games into the regular leisure activities of the residential care centre.

## Materials and methods

We included residents living in a residential care centre. Inclusion criteria were: able to stand up independently and walk at least 3 m, speaking and understanding Dutch, not suffering a serious medical condition that could pose a danger while playing exergames (e.g., epilepsy), performing a score of more than 20 on the Mini Mental State Examination.

With the aid of the staff members, we recruited and informed residents for participation. The GPs were informed about inclusion of their patients and a refusal of the GP to participate led to exclusion of the resident. For convenience reasons, the residents were included by unit where they resided. These units were randomly (supported by randomization software) allocated to the intervention arm and the control arm. The staff members were blinded for the intervention.

For the primary research question, we also questioned (by hetero-anamnesis) nine staff members: nurses, care assistants, and occupational therapists. We engaged eleven coaches to supervise the exergames and to complete the questionnaires related to the secondary research question: physiotherapists, occupational therapists, and animators.

The intervention took place during 13 weeks where exergames (boxing, bowling, football, etc.) with an Xbox 360 Kinect Sport were played in three residential care centres in Flanders. The intervention group played exergames twice a week for 13 weeks in sessions of 1 h per four people. These intervention group residents also had the opportunity to participate in other planned activities. The control group only participated in the planned activities (usual care). Before the start of the study, an introduction moment with hands on training was organised for the coaches in each residential care centre. Residents were informed by the staff members and through flyers. Before each game session, the coaches explained and demonstrated the game and the gear.

The primary research question was investigated via a questionnaire composed by merging selected questions from validated questionnaires with statements assessing sleep, mental wellbeing, pain complaints, and cognitive functioning (Table 1). The questionnaire was administered before the start (time point 0), after the completion of the exergames (3 months), and 6 months after the start of the study to residents from the intervention and control groups. Statements assessing mental wellbeing addressed the subdomains psychosocial wellbeing and autonomy. To assess sleep quality, the different stages of sleep such as falling asleep, staying asleep and daytime energy levels were questioned. To assess pain complaints, the statements addressed the frequency of the pain complaints and the impact of pain on general wellbeing. In total, 26 statements were submitted and scored on a Likert scale (0 = fully disagree; 5 = fully agree). To assess cognitive functioning memory six subjective statements, two recall questions, the clock-drawing test and the Stroop colour-word test were performed [16, 17].

**Table 1:** Composition of questionnaire by outcome and source

26 questions/statements	Source (*n* = number of questions)
Six statements on sleep quality	Domus Medica guideline sleep disorders (9)
Ten statements on mental wellbeing	Bel Rai (11) OPQOL-35 (12) RAND 36 (13)
Four statements on pain complaints	Bel Rai (10)
Six statements + two questions + two tasks to assess cognitive functioning	IQCODE-N (14) NHG guidelines dementia (15)

A nurse, care provider or occupational therapist (staff members) completed a similar questionnaire with ten statements about the sleep quality, cognition, pain complaints, and mental wellbeing of the residents (scored on a Likert scale from 0 to 5 ranging from ‘very bad’ to ‘very good’). To compile this questionnaire, the same sources were used as for the questionnaire of the residents.

In answer to the secondary research question, a coach kept a diary per participant during the 3-month test period for both the intervention and control groups. This diary also registered individual participation to the exergame session and the reason for any absence. In both the intervention and control group, participation in planned animation or regular activities was registered.

Coaches registered the fun factor on a VAS scale (from 0 to 10) [18]. The intensity of exercise was measured by the Borg scale (from six, very light effort to twenty, or maximum effort) [19]. In addition to this diary, a resident’s questionnaire assessed at the end of the 3 months the fun factor, social interaction, advantages and disadvantages and if the participant would still participate in the exergames in the future. All these outcomes were measured with a five-point Likert scale ranging from ‘fully disagree’ to fully agree’. Finally, a similar questionnaire for coaches assessed their experiences with playing exergames in residential care centres: was this feasible, what was the fun factor for coaches and what are the benefits and drawbacks?

Data analysis was limited to simple descriptive analysis since the study sample was expected to be small.

### Ethical approval

The staff informed residents about the intervention. If residents showed interest and agreed being approached for inclusion, the researchers further explained the study. For this purpose, the researchers used an informative flyer and a comprehensive information letter. If residents agreed to participate, they signed an informed consent. All methods were carried out in accordance with relevant guidelines and regulations and prepared in discussion with scientific and field experts. The Medical Ethical Board of the University Hospitals of KU Leuven approved the study (MP004468).

## Results

The intervention group started with 18 and the control group with 17 residents with an unequal distribution of gender (10 male versus 22 female) (Table 2). After 3 months there was a drop-out of three residents in the intervention group due to not interested anymore, back pain and too burdened.

**Table 2:** Characteristics of residents

Characteristics	Total (*n* = 32)	Intervention group (*n* = 15)	Control group (*n* = 17)
Gender			
Male	10	6	4
Female	22	9	13
Age			
< 76j	3	2	1
76–80j	3	0	3
> 80j	26	13	13
Education			
education until 14y	13	7	6
education until 16y	7	4	3
education until 18y	8	3	5
education until > 18y	3	1	2
Duration of stay			
1j	11	6	5
1–3j	14	6	8
> 3j	7	3	4
Walking aid			
None	8	3	5
Stick	1	0	1
Walker	21	12	9
Wheel chair	2	1	1
Marital status			
Not married	3	0	3
Married	9	4	5
Divorced	2	2	0
Widow(er)	18	9	9
Children			
0	5	2	3
1	6	1	5
2	4	2	2
> 2	17	10	7
Earlier sport			
Yes	17	7	10
No	15	8	7
Earlier video games			
Yes	4	1	3
No	28	14	14
Earlier exergames			
Yes	2	1	1
No	30	14	16

Primary research questions (Table 3).

**Table 3:** Effect of exergaming versus usual care on wellbeing, sleep, pain, and cognition

Median score on questionnaire	Intervention group Time point			Control group Time point		
	0 m	3 m	6 m	0 m	3 m	6 m
Wellbeing rated by residents/50	42	44.5	45	41	41	41
Wellbeing rated by staff/20	18	18	16	14	17	16
Sleeping score rated by residents/30	23	24	28	24	25	25
Sleeping score rated by staff/10	7	7.5	8	7	7	7
Pain score rated by residents/20	18	18.5	20	17	14	16
Pain score rated by staff/op5	4	4	4	4	4	4
Cognitive function rated by residents/30	24	26	26	23	27	26
Cognitive function rated by staff/30	13.5	13	13	12	11	11
(Mean) Cognitive function objective/2	1.8	1.9	1.7	1.2	1.6	1.5
(Mean) Clock drawing test/5	3.1	2.9	3.5	2	1.8	2.5
Stroop colour-word test in seconds	28	29.5	28	37	30.5	30.5

The median mental wellbeing score (of maximum 50) for the residents of the intervention group at time points 0, 3, and 6 months was 42, 45, and 45, respectively. For the residents of the control group, this score was 41 at all three time points. The median score (of maximum 20) of the staff member questionnaire on the mental wellbeing of the residents was 18, 18, and 16 in the intervention group at the respective time points of 0, 3, and 6 months. The scores of the control group at the same time points were 14, 17, and 16, respectively.

The median sleep score (of maximum 30) for the residents of the intervention group at time points 0, 3, and 6 months was 23, 24, and 28 respectively. For the residents of the control group this was 24, 25, and 25, respectively. The median score (of maximum 10) of the staff member questionnaire on the sleep quality of the residents was of 7, 7.5, and 8 in the intervention group at the respective time points of 0, 3, and 6 months. The scores of the control group remained equal to seven over the full 6 months.

The median pain score (of maximum 20) among the residents of the intervention group at time point 0, 3, and 6 months, respectively, was 18, 18.5, and 20. For the residents of the control group this was 17, 14, and 16, respectively. The median score (maximum of 5) of the staff member questionnaire about the pain complaints of the residents was four at all-time points in both the intervention and control groups.

In the intervention group, the median score (maximum of 30) on subjective cognition was 24, 26, and 26 at the respective time points. In the control group, this score was 23, 26, and 27. The mean scores (maximum of 2) on the two open questions assessing cognitive functioning of the residents evolved from 1.8 at the start to 1.9 and to 1.7 after 3 and 6 months, respectively, in the intervention group. In the control group, these scores were 1.2, 1.6, and 1.5 at the same time points. The mean scores (maximum of 5) on the clock-drawing test, evolved from 3.1 at the start to 2.9 and to 3.5 after 3 and 6 months, respectively. In the control group, these scores were 2; 1.8 and 2.5 at the same time points.

In the Stroop-color-word test, the median time difference between correctly naming the colors of column 2 and column 3 in the intervention group was 28 s, 29.5 s, and 28 s at time points 0, 3, and 6 months. In the control group, these median time differences were 37 s, 30.5 s, and 30.5 s at the same times.

### Secondary research questions

On average, 23.3 exergame sessions were organised in the three residential care centres. As registered by the coaches, there was an average participation rate of 88%. The main reasons for not showing up were other animation, visits of relatives and physical complaints. Coaches gave an average fun score of 8.9/10 and an average intensity of exercise score of 11.6/20 over the three months of playing (Table 4).

**Table 4:** Average fun and intensity score in exergaming group

Average score	Intervention group/time points		
	0 m	3 m	6 m
VAS (fun)	8.7	8.9	9
Borg (intensity)	10.9	11.6	13.1

After 3 months, residents were asked if they had fun, if they made new friends and if they would continue to play it in the future. On a five-point Likert scale, the average of these responses were 4.4, respectively; 2.8 and 4.4. Residents particularly liked being together and playing the games. Residents mentioned that feeling fitter and upgrading their social contacts were the major advantages of the exergames. For future exergame sessions, residents suggest offering more different games, organizing a weekly session and playing together with several people (Table 5).

**Table 5:** Advantages and disadvantages of exergames reported by residents

	Number of answers
Advantages	
Feeling fitter	5
Social contact	2
Being busy	2
Other than usual activity	1
Competition	1
New sport	1
Disadvantages	
Pain arms or back	3
Fear of falling	1

According to the coaches, the feasibility of the exergames and the positive effect both scored on average 4.1 on a five-item Likert scale. The average score on ‘continue playing in the future’ was 4.3 and on ‘fun factor for the coaches’ 4.7. The major advantages according to the coaches were maintaining mobility, the fun factor and the sportive, competitive ambience. Major disadvantages were the low accessibility for more physically disabled residents, the intensive coaching and the limited offer of games. The coaches advised to develop games specifically serving elderly (Table 6).

**Table 6:** Positive effects, advantages, disadvantages, and suggestions for future reported by coaches

	Number of answers
Positive effect	
Fun	3
Competition	3
Innovative	3
Motivated residents	2
Ground-breaking	1
Integration in care centre	1
Recall of sports, memories	1
Advantages	
Exercising	5
No extra material investment, easy to apply	3
Social contacts	2
Bringing seniors in contact with ‘modern times’	1
Playing with family/visitors	1 1
Disadvantages	
Low accessibility for disabled residents	3
Coaching	3
Limited variation in games	3
Games not always intuitive	2
Exclusion of residents	1
English	1
Future	
Games for seniors	4
Lower frequency	3
More inclusive	1
Not playing in warm season	1

## Discussion

In this study, we observed an improvement of self-reported mental wellbeing, sleep pain complaints and cognition during exergaming and at 6 months follow up. An objective assessment of cognition, however, did not reveal a sign of improvement. Staff members reported an improved mental wellbeing in the control group.

The fun factor and the intensity of exercise reported by the coaches were high. They also appreciated the feasibility of exergaming and the positive impact on the residents although they regretted the limited accessibility for disabled residents and the intensity of supervision. Residents also mentioned feeling, fitter, having fun playing and they would like to continue playing if organised on a structural base. They did not claim meeting new friends but they appreciated the social aspect of exergaming.

Residents reported a positive effect of gaming on their mental health better but staff members did not notice this change. The improvement in mental wellbeing of the gaming residents was in accordance with findings in comparable research [4]. It is unclear if this effect is due to exergaming or to the mere impact of a new activity [12]. Indeed, the gaming residents particularly appreciated the social contacts and the grind breaking impact of this very new game. The Xbox and games remained available to the residential care centre for an additional 3 months after ending the study, residents from the intervention group were able to game on a voluntary basis which might also explain the lasting positive effect.

Physical activity during the day is a known sleep-promoting factor what might explain the improvement of sleep quality [14, 20]. After 6 months, we see an additional increase in the sleep score among the residents of the intervention group. These residents were still able to exergame, which might explain the lasting positive effect on sleep, despite playing less intensively and without supervision.

As in other studies, there was an overall improvement in the pain complaints in the intervention group compared to the control group [21]. Physical exercise has a beneficial effect on various pain complaints [22]. Pain complaints are also very variable over time and context dependent. It is assumable that musculoskeletal pain emerges when sedentary residents suddenly start exercising [5, 23].

The results did not show a convincing influence of exergames on the cognitive functioning of the residents. These experiences contrast with results from other research [8, 9, 23, 24]. A meta-analysis showed that exergaming positively affected attention, the visuospatial ability and executive functions [8]. A 2016 systematic review also showed promising results in terms of cognition after playing exergames [25]. In our study, residents were free to participate any time and they did not play on a scheduled base. Second, the games were in particularly focusing on physical exercise. Finally, we included a sample of cognitively well-functioning residents. A structured schedule of playing, with games focusing on cognitive competences and applied to a cognitively frail group, might have a more positive effect on cognition [10, 25].

Coaches and participants appreciated the fun factor and the social interaction during exergaming. Similar results were reported in other research [4, 9, 23]. Mental and physical wellbeing are linked to feelings of pleasure and to social contact and we can assume that this should be the major goal of exergaming or any other activity in a residential care centre. Beside, to maximize the positive effects of exergaming and to remain attractable over time, coaches should consider scheduling playtime and to offer more gaming variation. To increase accessibility, an offer of less physically demanding exergames should be available wherefore also more disabled residents can participate [3, 26].

The main limitation of this study was the small study population, obstructing in depth statistical analyses. Any inclusion bias cannot be ruled out: to play exergames residents needed to be fit and mentally resilient. However, even with this relatively competent group of participants we observed improvements in wellbeing. Second, the blinding was not always successful since residents shared their participation in the study to the blinded staff. Third, to control the time spent to complete the questionnaires, we compiled a short version of validated instruments. Due to the small study sample, a validation of these questionnaires was impossible.

A strength of the study is that implementation of the exergames fitted in the usual offer of activities which facilitates transfer of the procedure and the results ta daily practice. Second, the exergames were easy and intuitive to use, lowering the time investment and threshold for playing and coaching. Third, the study sample was larger than reported in other studies in this domain and context.

## Conclusions

This randomized study in three Flemish residential care centres showed that playing exergames positively affected general wellbeing defined as sleep quality, mental wellbeing, and pain complaints Cognitive functioning did not improve after the introduction of exergames but this might be related to the cognitively strong group of participants. Over time, the residents enjoyed the exergames and the social contact while they also felt fitter. The coaches appreciated the feasibility of the exergames and they believed implementation in the usual care of a residential care centre requires variation in games and a scheduled programme. Exergaming is an attractive and feasibly complement to the usual activities offered in a residential care setting. Future research should focus on more physically and mentally vulnerable groups and should aim to develop a study in an implementation design.

## Data Availability

The datasets generated and/or analyzed during the current study are available in the link: https://drive.google.com/drive/folders/10clsG3acKRJr3m8LvD0L4FxVOwr5I0R_?usp=sharing

## Declarations

### Human and rights statement

The Medical Ethical Board of the University Hospitals of KU Leuven approved the study (MP004468).
